# A Retrospective Study of Foreign Body Ingestions and Outcomes Among the Pediatric Age Group in a Tertiary Care Hospital in Dubai, United Arab Emirates

**DOI:** 10.7759/cureus.88229

**Published:** 2025-07-18

**Authors:** Dana Sarmini, Mohammed O Ibrahim, Balaji Krishnamurthy

**Affiliations:** 1 Pediatrics, Al Jalila Children's Specialty Hospital, Dubai, ARE; 2 College of Medicine, Mohammed Bin Rashid University of Medicine and Health Sciences, Dubai, ARE; 3 Pediatric Gastroenterology, Al Jalila Children's Specialty Hospital, Dubai, ARE

**Keywords:** button battery ingestion, coin ingestion, esophageal impaction, foreign body ingestion, magnet ingestion, pediatric emergency

## Abstract

Background

Foreign body ingestion (FBI) is a prevalent pediatric emergency, especially in toddlers. The clinical trajectory is significantly influenced by the type, size, and location of the object. Although many cases resolve without intervention, high-risk foreign bodies, including button batteries and magnets, present considerable health risks. Limited data are available from the United Arab Emirates (UAE) and the Gulf region.

Objective

This study aimed to evaluate the clinical characteristics, management strategies, and outcomes of pediatric FBI cases presenting to Al Jalila Children's Specialty Hospital over a 15-month period in addition to look for secondary outcomes that help with reducing the complication rate.

Methods

This retrospective cross-sectional study included all pediatric patients (aged 0-18 years) presenting with confirmed or suspected FBI between December 2022 and February 2024. Data were extracted from electronic medical records, including demographics, clinical presentation, foreign body type, number, and location, time from ingestion to emergency department (ED) presentation, management modality, and complications. Statistical analysis, including descriptive statistics and chi-squared tests, was conducted to determine associations between object type and rates of intervention and complications.

Results

A total of 338 cases were included. The mean age at presentation was 4.35 years (±3.26), ranging from six months to 16 years, with 202 (59.8%) being male. Coins were the most commonly ingested object, seen in 127 (37.6%) cases, followed by magnets in 40 (11.8%), button batteries in 37 (10.9%), and plastics in 31 (9.2%). While 76 (22.5%) patients required some intervention (endoscopy or surgery), 262 (77.5%) were managed conservatively. Ingestion of button batteries and multiple magnets required significantly higher intervention with higher complication rates. A statistically significant association was found between foreign body type and the need for intervention (χ² = 29.71; p < 0.001), as well as complication occurrence (χ² = 22.71; p < 0.001). In addition, the overall complication rate was 11 (3.3%), primarily involving button batteries.

Conclusion

This study examines a cohort of pediatric patients presenting with FBI at a single tertiary care center in Dubai. The condition predominantly affects young children, with coins being the most frequently ingested items. However, ingestion of high-risk objects, such as magnets and button batteries, poses significantly greater dangers, often necessitating immediate medical intervention. These findings underscore the urgent need for enhanced public awareness, streamlined referral pathways, and the development of national guidelines tailored to the region's unique clinical trends.

## Introduction

Foreign body ingestion (FBI) is one of the most common pediatric emergencies worldwide, predominantly affecting children under five years. Children in this age group have a strong oral exploration drive, frequently mouthing objects, and are highly inquisitive, which can contribute to young children swallowing small non-food items [1]. FBI is responsible for a large proportion of visits to the pediatric emergency department (ED) and can have a broad clinical spectrum, from asymptomatic cases to potentially fatal complications such as esophageal perforation, aortoesophageal fistula, or sepsis [2]. The esophagus is the most common site of foreign body impaction, especially at its physiologic narrowing [3]. While most ingested foreign bodies pass through spontaneously, specific types, notably coin batteries, sharp metallic objects, and strong magnets, are associated with a high risk of morbidity and frequently necessitate urgent endoscopic or surgical interventions [4].

Initial manifestations of pediatric FBI are usually nonspecific. Symptoms can include drooling and salivation, dysphagia, vomiting, thoracic pain, cough, or agitation [5]. In most cases, children do not exhibit symptoms or are unable to describe them, making early diagnosis challenging. Imaging studies are instrumental in diagnosing, especially when the object is radiopaque (e.g., batteries, coins, nails). Nevertheless, if clinical suspicion is absent, synthetic polymers, such as plastics or biological materials, may not be identified [6].

The type of foreign body consumed is a significant component in determining the clinical course. Coins are the most commonly consumed objects by kids, particularly in Western countries, according to multiple studies [7]. Button batteries are particularly dangerous as they can produce hydroxide ions, causing liquefactive necrosis within hours of ingestion [8]. Alkaline damage to the esophagus can persist even after battery removal. Severe problems have been recorded, such as tracheoesophageal and aortoesophageal fistulas, 2-3 weeks post-button battery removal from the esophagus [9]. Ingesting numerous magnets or one magnet with a metallic instrument can cause intestinal loop entrapment, necrosis, and perforation [10].

To address these risks, international guidelines, such as those issued by the North American Society for Pediatric Gastroenterology, Hepatology, and Nutrition (NASPGHAN) and the European Society for Pediatric Gastroenterology, Hepatology, and Nutrition (ESPGHAN), have outlined evidence-based algorithms for managing foreign bodies. These guidelines prioritize the early endoscopic removal of button batteries in the esophagus, the rapid assessment of multiple magnet ingestion, and weighing the observation versus intervention decision depending on the location and type of object [11].

Although the global burden of pediatric FBI is well-documented, there remains a paucity of region-specific literature from the Arab world and the Gulf Cooperation Council (GCC) countries. In a retrospective study from Saudi Arabia, Abudungor et al. analyzed 244 pediatric cases of FBI and found that coins were the most commonly ingested item, with the stomach being the most frequent site of impaction. Over half of the cases required endoscopic removal, and approximately 4% developed complications, including strictures and mucosal erosions [12]. In another study from Makkah, Saudi Arabia, coins were also identified as the most common foreign body type, while button batteries and sharp objects were widely accepted as the most clinically significant. They emphasized the importance of public education and early medical attention in reducing these rates of complications [13].

In Oman, a retrospective study by Al Lawati and Al Marhoobi examined 134 cases of FBI, with 60% of cases undergoing intervention, resulting in a relatively low complication rate due to prompt presentation and management [14]. Data on this are limited from Kuwait and Bahrain, but case reports and institutional audits suggest similar trends in these countries, with coins, batteries, and toy parts being the most common items seen in clinical practice.

Despite these regional insights, a significant gap remains in the literature from the United Arab Emirates (UAE). To date, only one published study from Dubai by Farouk and AlSuwaidi has focused exclusively on toxicological (chemical and pharmaceutical) ingestion rather than mechanical FBI, such as coins, button batteries, or magnets [15]. Although this was a hospital-based study, it did not cover gastrointestinal FBI, and an important aspect was missing from the UAE's clinical literature. Consequently, data on non-toxic foreign bodies ingested and their anatomical location, clinical presentations, and outcomes are limited. This shortcoming hinders ED and pediatricians in the UAE from comparing practices with those in the region and internationally or adapting management protocols to local epidemiology.

Given the rapid growth of the pediatric population in Dubai and its highly heterogeneous nature due to the multinational demographics, it is essential to understand the local patterns of FBI. Based in Dubai, Al Jalila Children's Specialty Hospital, the sole dedicated pediatric tertiary care center in the emirate, is uniquely positioned to provide rich datasets that accurately capture the clinical realities of the UAE. Considering that no evidence exists in the form of national registries or prospectively designed large cohort studies from the region to compare with this study, hospital-based studies are important to know the burden of disease in the region, which can help generate guideline-based evidence. This study aims to address the current lack of local data on mechanical FBIs in children within the UAE. We retrospectively analyzed all pediatric cases presenting to Al Jalila Children's Specialty Hospital over a 15-month period to evaluate the type and anatomical location of ingested foreign bodies, patient demographics, clinical presentations, management strategies, and associated complications. The findings aim to inform evidence-based national guidelines and guide public health interventions tailored to the region's unique pediatric population. 

## Materials and methods

Study design and setting

This was a single-center, retrospective, cross-sectional study conducted at Al Jalila Children's Specialty Hospital, a tertiary referral center and the only specialty hospital for pediatrics in the UAE. The center's unique status provides an extensive experience with pediatric emergencies, where the impact and outcomes of FBI could be assessed in children. Data were collected for pediatric patients who presented with FBI over a 15-month period, from December 2022 to February 2024. Ethical approval was granted by the Mohammed Bin Rashid University of Medicine and Health Sciences Institutional Review Board (MBRU-IRB) (approval number: MBRU IRB-2024-197).

Study population

The study included all pediatric patients under the age of 18 years who presented to the ED with suspected or confirmed solid FBI, identified through clinical assessment and/or imaging.

Inclusion criteria

The inclusion criteria included all children under the age of 18 years who presented with a history or confirmation of FBI. Eligible cases included the ingestion of solid, non-toxic objects such as coins, magnets, button batteries, toy parts, and other similar items. Patients with trichobezoar ingestion were also included. 

Exclusion criteria

The exclusion criteria included patients aged 18 years or older and cases involving foreign body aspiration into the respiratory tract. Additionally, caustic (chemical) ingestions were excluded due to their distinct pathophysiological mechanisms and management pathways. 

Data collection and variables

Clinical data were extracted from the hospital's electronic medical record (EMR) system by the study team and entered into a pre-structured Microsoft Excel sheet (Microsoft Corp., Redmond, WA, USA). Cases were identified through diagnostic code filtering and keyword searches related to FBI. The collected variables included demographic data (age and sex), clinical presentation (symptoms such as vomiting, dysphagia, drooling, and abdominal pain, presence of comorbidities, and time until arrival to the ED), details of the foreign body (type, number, and anatomical location, i.e., esophagus, stomach, or intestines), management approach (need for observation, endoscopy, or surgery), and outcomes, including complications such as perforation, stricture formation, mucosal erosion, or necrosis. Cases involving button battery ingestion were monitored closely due to their known potential for rapid and severe complications, including liquefactive necrosis and the risk of tracheoesophageal or aortoesophageal fistula. All data were anonymized to protect patient confidentiality, and access to the dataset was restricted to the study investigators. 

Sample size

This study used total population sampling; all eligible cases presenting within the 15-month period were included. Based on the preliminary screening of records, the expected sample size was 338 cases, sufficient to allow subgroup analyses by foreign body type and outcome.

Data analysis

All statistical analyses were performed using IBM SPSS Statistics for Windows, V. 26.0 (IBM Corp., Armonk, NY, USA). Descriptive statistics were used to summarize the data: continuous variables, such as age, were reported as mean ± standard deviation (SD), while categorical variables, including gender and foreign body type, were expressed as counts and percentages. For inferential analysis, the chi-squared test was employed to examine associations between foreign body type and key outcomes such as complication rates and the need for intervention. A p-value of less than 0.05 was considered statistically significant. No imputation methods were applied; cases with missing data were excluded from the relevant analyses.

Reporting standards 

This study is reported in accordance with Strengthening the Reporting of Observational Studies in Epidemiology (STROBE) guidelines for cross-sectional studies. 

## Results

A total of 338 pediatric patients with FBI were included in the analysis, all of whom met the inclusion criteria and had no missing data for the primary variables. The mean age at presentation was 4.35 years (±3.26), ranging from six months to 16 years (Table 1). Nearly half of the patients, 149 (44.1%), were between one and three years old, followed by 96 (28.4%) in the 4-6-year age group and 75 (22.2%) aged seven years or older. Infants under one year comprised 18 (5.3%) of the study population. Males constituted 202 (59.8%) of cases, while females comprised 136 (40.2%) (Table 1). Most patients, 320 (94.7%), had no underlying comorbidities; 18 (5.3%) had other disorders, including eosinophilic esophagitis and neurological disorders. 

**Table 1:** Demographic characteristics of pediatric patients with foreign body ingestion

Variable	Number (%)
Age group	
<1 year	18 (5.3%)
1-3 years	149 (44.1%)
4-6 years	96 (28.4%)
≥7 years	75 (22.2%)
Sex	
Male	202 (59.8%)
Female	136 (40.2%)
Comorbidities	
Present	18 (5.3%)
Absent	320 (94.7%)

**Figure 1:**
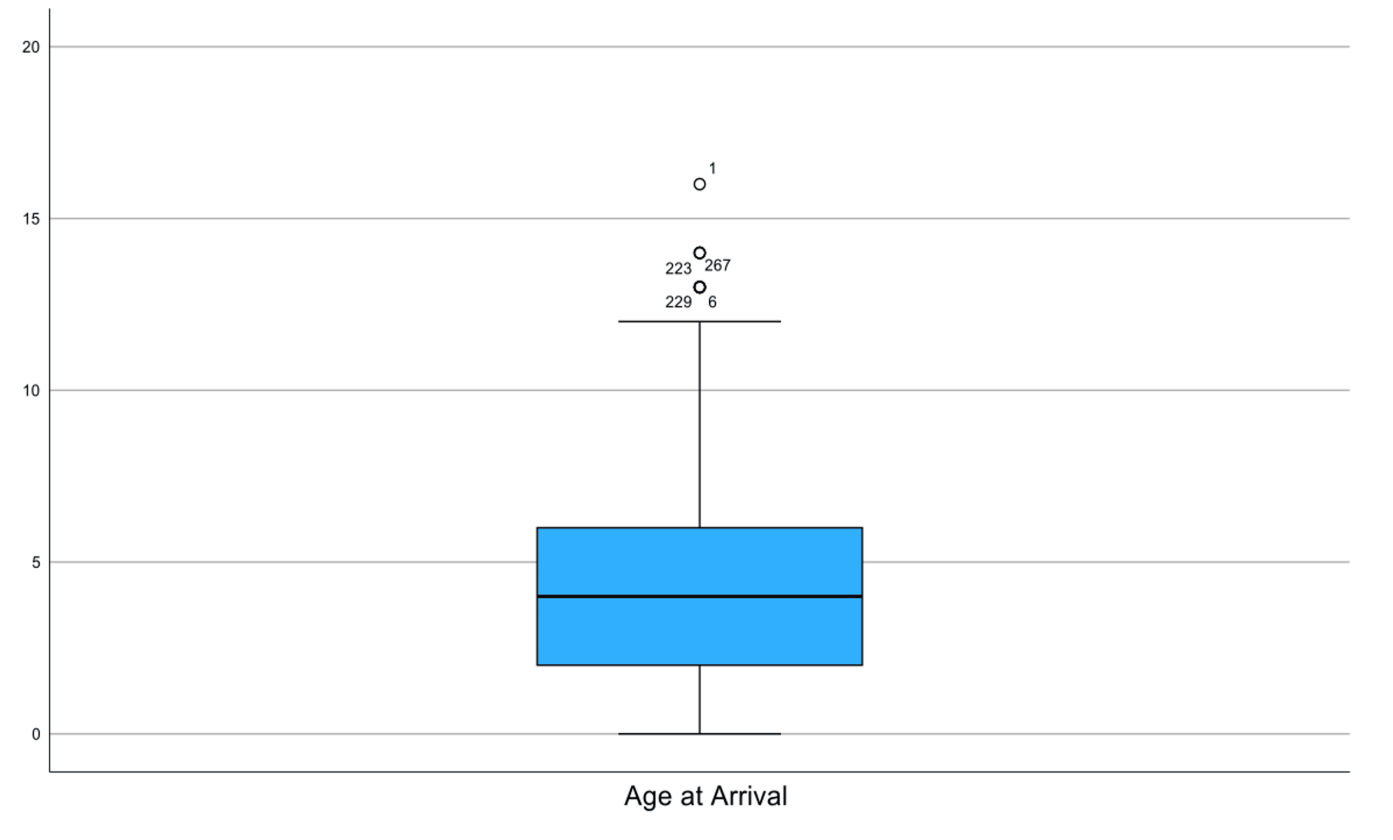
Box plot of patient age at arrival showing a right-skewed distribution with clustering in early childhood The "°" symbol indicates statistical outliers beyond the interquartile range.

The full age distribution is presented in Figure 1, which displays a box plot illustrating a right-skewed age distribution, with the majority of cases occurring between one and six years. A few older children present as outliers above 12 years. The positive skew toward early childhood.

The majority of children were asymptomatic at the time of presentation, 224 (66.3%), with the most commonly reported symptoms among the remainder being drooling in 40 (11.8%), nausea or vomiting in 29 (8.6%), abdominal pain in 25 (7.4%), and dysphagia in 19 (5.6%) (Table 2).

**Table 2:** Presenting symptoms at the emergency department

Symptom	Number (%)
Asymptomatic	224 (66.3%)
Drooling	40 (11.8%)
Nausea/vomiting	29 (8.6%)
Abdominal pain	25 (7.4%)
Dysphagia	19 (5.6%)
Constipation	1 (0.3%)

**Figure 2:**
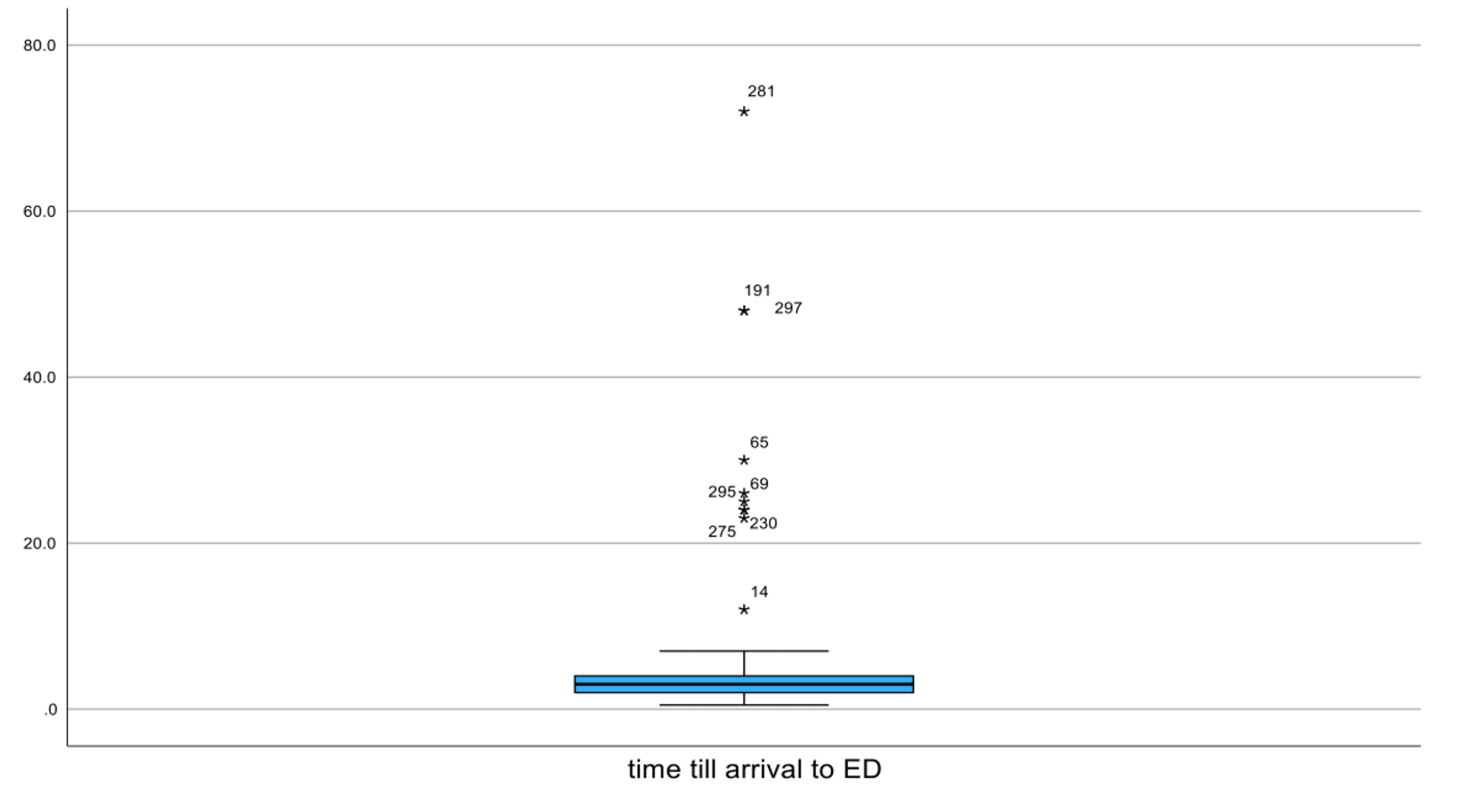
Box plot showing time from ingestion to emergency department presentation (hours) Asterisks (*) indicate extreme outliers, defined as values more than three times the interquartile range above the upper quartile.

Figure 2 displays the time elapsed between FBI and presentation to the ED. The distribution is strongly right-skewed, with most patients presenting within the first few hours of admission. The median time to presentation is low, but several outliers are visible, with several patients arriving more than 20, 40, and even 70 hours post-ingestion. These delayed cases are primarily explained by patients initially presenting to other healthcare facilities and being referred for evaluation at the tertiary hospital.

Coins were the most commonly ingested foreign objects, accounting for 127 (37.6%) of cases, followed by metal items in 54 (16%), magnets in 40 (11.8%), button batteries in 37 (10.9%), and plastic objects in 31 (9.2%). Less frequently ingested items included toy parts, food boluses, trichobezoars, and sharp objects (Table 3). Among the magnets ingested, 28 (71%) were high-powered neodymium magnets, and 12 (29%) were traditional magnets; notably, 14 (35%) of these incidents involved multiple magnets. While the majority of patients, 317 (93.8%), ingested a single object, 21 (6.2%) presented with multiple ingestions, including one child who had swallowed 16 individual items.

**Table 3:** Types of foreign bodies ingested by patients

Type of foreign body	Number (%)
Coin	127 (37.6%)
Metal object	54 (16%)
Magnet	40 (11.8%)
Other (toys/unknown objects)	38 (11.2%)
Battery	37 (10.9%)
Plastic object	31 (9.2%)
Food bolus	8 (2.4%)
Trichobezoar	2 (0.6%)
Sharp object	1 (0.3%)

Radiological or endoscopic findings revealed that the stomach was the most common site of impaction, identified in 117 (34.6%) cases, followed by the intestines in 94 (27.8%) and the esophagus in 59 (17.5%). In 68 (20.1%) patients, the foreign body was not visualized on imaging, though suspicion remained high based on clinical history and examination, as shown in Table 4. 

**Table 4:** Anatomical location of foreign body based on imaging or endoscopy

Location	Number (%)
Stomach	117 (34.6%)
Intestines	94 (27.8%)
Esophagus	59 (17.5%)
Not visualized	68 (20.1%)

In terms of clinical management, 76 (22.5%) patients required active intervention, with 72 (21.3%) undergoing endoscopy and four (1.2%) requiring surgical procedures. The remaining 262 (77.5%) were successfully managed through conservative, non-invasive approaches. A statistically significant association was observed between the type of foreign body ingested and the likelihood of intervention (χ² = 29.71; p < 0.001). High-risk objects such as button batteries, coins, and magnets were significantly more likely to necessitate endoscopic or surgical removal. Specifically, 10 (27%) button battery cases underwent endoscopy, while 27 (73%) were managed conservatively. Among coin ingestions, 36 (28.3%) required endoscopic extraction, with 91 (71.7%) treated non-operatively. Magnet-related cases revealed that 11 (27.5%) required endoscopic removal and an additional three (7.5%) necessitated surgical intervention, particularly in cases involving the ingestion of multiple magnets simultaneously (Table 5).

**Table 5:** Clinical management and intervention by foreign body type χ² (df = 6); χ² = 29.71; p < 0.001

Foreign body type	No intervention	Endoscopy	Surgery	Total cases	% with intervention
Battery	27 (73%)	10 (27%)	0 (0%)	37	27%
Coin	91 (71.7%)	36 (28.3%)	0 (0%)	127	28.3%
Magnet	26 (65%)	11 (27.5%)	3 (7.5%)	40	35%
Others	118 (88.1%)	15 (11.2%)	1 (0.7%)	134	11.9%
Total	262 (77.5%)	72 (21.3%)	4 (1.2%)	338	22.5%

Complications were reported in 11 (3.3%) of cases. Of these, eight (2.4%) experienced short-term complications, such as mucosal erosion or inflammation, one (0.3%) developed long-term complications, including strictures that required multiple follow-ups and dilatations, and two (0.6%) had both short- and long-term complications. Most of these complications were associated with button batteries and, to a lesser extent, magnets. Statistical analysis revealed a significant association between the type of foreign body and the likelihood of complications (χ² = 22.71; p < 0.001), with button batteries accounting for six (16.2%) of all complications compared to just one (0.8%) for coins and one (2.5%) for magnets (Table 6). 

**Table 6:** Association between type of foreign body and clinical complications χ² (df = 3); χ² = 22.71; p < 0.001

Foreign body type	No complication	Complication	Total (n)	% with complication
Battery	31 (83.8%)	6 (16.2%)	37	16.2%
Coin	126 (99.2%)	1 (0.8%)	127	0.8%
Magnet	39 (97.5%)	1 (2.5%)	40	2.5%
Others	131 (97.8%)	3 (2.2%)	134	2.2%
Total	327 (96.7%)	11 (3.3%)	338	3.3%

**Figure 3:**
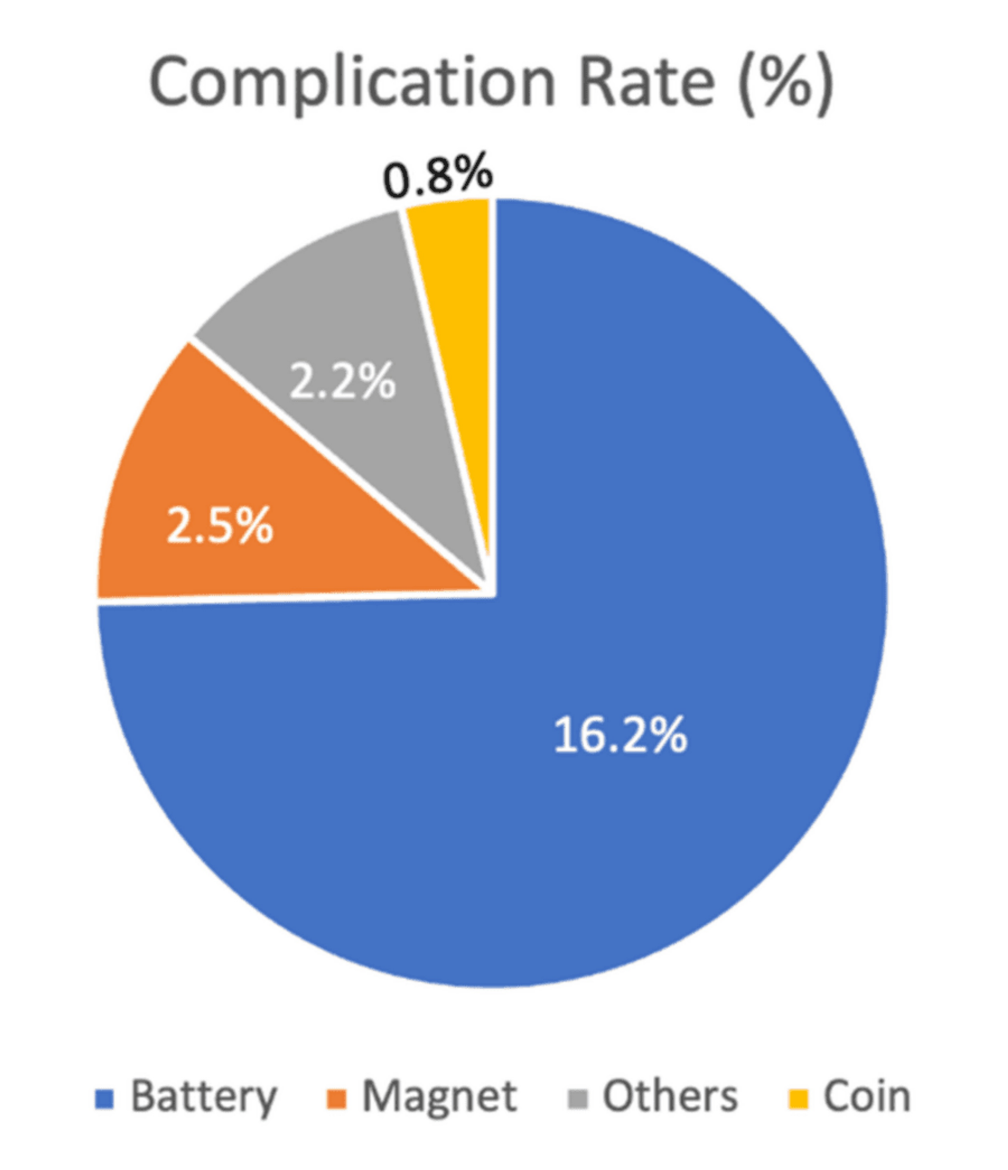
Distribution of complications by foreign body type (%)

There was no statistically significant association between gender and complication rates (χ² = 2.339; p = 0.505). Overall, the findings suggest that while most pediatric FBIs are managed without complications, certain object types, particularly button batteries and magnets, carry a significantly higher risk of requiring intervention and causing harm. Button batteries accounted for the largest proportion of complications, with six (16.2%), followed by magnets (one, 2.5%). Complications associated with coins and other objects were rare, reflecting lower clinical risk (Figure 3). 

## Discussion

This retrospective cross-sectional study provides insight into the clinical features, diagnostic approaches, management strategies, and outcomes of pediatric FBI in the UAE. Our results align with regional and international data, highlighting that the most vulnerable age group is between one and three years old due to their exploratory behavior and limited risk perception [16]. In this developmental phase, oral investigation of objects is a typical activity that predisposes children to accidentally ingesting small household items [17].

Coins were the most commonly ingested object in our cohort, mirroring global patterns in which coins are the leading cause of FBI in children due to their ubiquity and accessibility [18]. However, batteries and magnets, although less frequent, were significantly more dangerous. Button batteries, in particular, pose a well-documented risk of causing severe injury through electrochemical burns, tissue necrosis, and vascular erosion when lodged in the esophagus [19]. Within two hours of mucosal contact, these batteries can generate hydroxide ions at the negative pole, resulting in liquefactive necrosis [20]. Numerous case reports and observational studies have described outcomes ranging from tracheoesophageal fistulas to fatal hemorrhage from aortoesophageal fistulas following delayed recognition and removal [21].

Our results support these findings, demonstrating a significant association between foreign body type and the need for invasive management (χ² = 29.71; p < 0.001). Endoscopy was required in 72 (21.3%) of cases overall, and surgical intervention in four (1.2%). Button batteries and magnets were significantly more likely to require such interventions. Notably, 10 (27%) button battery cases and 14 (35%) magnet cases led to endoscopic or surgical procedures. Magnet ingestion cases that required intervention were only needed when multiple magnets were ingested, highlighting their higher clinical risk profile due to magnetic attraction across bowel walls, causing pressure necrosis. These trends align with the guidance of the NASPGHAN, which recommends the urgent endoscopic removal of esophageal button batteries and close observation or removal of multiple magnets [11,22].

While most children were brought to care shortly after swallowing a foreign object, a notable few arrived only after more than 24 hours had passed. In many of these cases, families had initially sought help at clinics or hospitals that were not specialized in pediatric emergencies. Unfortunately, such delays can make treatment more challenging and raise the risk of serious harm, especially when button batteries or sharp items are involved. Other studies have described similar scenarios, often linked to gaps in caregiver understanding or limited awareness among first-contact health providers [23]. Strengthening early recognition through education and ensuring clear referral pathways can make a significant difference in providing high-risk children with the timely care they need.

Regarding anatomical distribution, imaging and endoscopic assessments revealed that the stomach was the most common site of object localization, followed by the intestines and esophagus. This finding is consistent with gastrointestinal transit patterns, in which small, smooth objects that pass through the esophagus tend to settle temporarily in the stomach [24]. While less common in our cohort, esophageal impaction is of higher clinical concern due to its proximity to vital structures and higher risk of mucosal injury. Importantly, in 68 (20.1%) patients, foreign bodies were not visualized radiologically. These cases often involved radiolucent objects such as plastic or rubber, underlining the importance of clinical judgment and symptom assessment even in the absence of imaging confirmation [22].

The overall complication rate in our cohort was 11 (3.3%), consistent with reported rates of 3-4% in multicenter regional studies [25]. Statistical analysis revealed a significant association between the type of foreign body and the likelihood of complications (χ² = 22.71; p < 0.001). Complications in this study were primarily associated with button battery ingestion, which involves mucosal erosions, suspected perforations, and strictures that require repeated intervention. The findings indicate that batteries present the most significant risk of adverse outcomes, with multiple magnets following in severity. By contrast, coins and other metallic or blunt objects rarely led to complications, consistent with their typically benign course [17,18].

This study offers several important strengths. The findings reflect the real-world patterns of FBI in a national pediatric population by capturing all eligible cases over a defined 15-month period at the UAE's only dedicated pediatric referral center. The use of total population sampling and consistent data extraction also strengthens the clinical and demographic reliability of the results.

From a public health standpoint, our results emphasize the significance of regulatory safety interventions and caregiver education. We recommend more effective labeling and safety designs for battery compartments, as well as required warnings on toys that include magnets and anticipatory counseling during well-child visits. These interventions have been shown to be effective in reducing intake rates and severity of outcomes [26]. Early recognition and prompt ED visits might be substantially enhanced by multilingual education campaigns aimed at various populations in a multicultural setting such as Dubai. Still, the study has its limitations. As with most retrospective designs, it was constrained by the quality and completeness of existing medical records. Information on symptom resolution, late complications, or follow-up outcomes was not always available, particularly for patients managed outside the hospital after discharge. Additionally, because this study was conducted at a single tertiary center in an urban setting, the results may not fully represent the experiences of individuals in more rural or resource-limited areas. 

## Conclusions

Our study aligns with international findings while providing valuable local data. Batteries and magnets, although ingested less frequently than coins, pose a far greater clinical risk and account for the majority of complications and interventions. Early identification, caregiver education, and timely referral remain critical for minimizing morbidity. These results support the development of national guidelines and public health policies specific to the epidemiology of FBI in the UAE and similar high-income, multicultural settings. 
